# Supporting paediatric patients to receive radiation therapy without sedation or general anaesthetic

**DOI:** 10.1002/jmrs.734

**Published:** 2023-10-25

**Authors:** Moira O'Connor, Georgia K. B. Halkett

**Affiliations:** ^1^ School of Population Health/enAble Institute, Faculty of Health Sciences Curtin University Bentley Western Australia Australia; ^2^ Curtin School of Nursing/Curtin Health Innovation Research Institute (CHIRI), Faculty of Health Sciences Curtin University Bentley Western Australia Australia

## Abstract

Many paediatric patients experience anxiety and distress when undergoing radiation therapy and, as a result, are often anaesthetised or sedated (A/S) so that they remain still. The practice of using A/S has implications for the child, the family and the health system. Building on the article by McCoola et al. (DOI 10.1002/jmrs.705), this editorial discusses approaches to improving paediatric patients' and their families' experiences of radiation therapy by reducing the need for A/S. Interventions need to be underpinned by theory and adopt robust research methods.

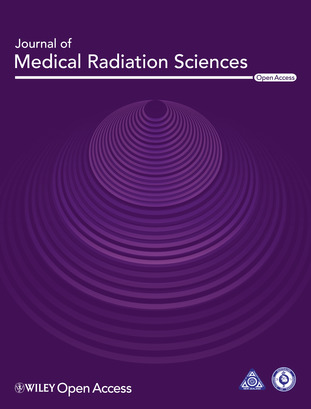

Radiation therapy is used in the optimum management of paediatric cancers,^1^, ^2^ with good outcomes, but there is the potential for adverse consequences.^3^ Unintended radiation to critical organs^3^ and the possibility of long‐term side effects mean that children must remain still for treatment.^4^, ^5^

When children are non‐compliant and cannot remain still, often due to anxiety and distress, a decision is often made to administer sedation/anaesthetic (S/A), usually a general anaesthetic (GA). However, daily S/A over a period of 6–7 weeks can have a negative impact on the child due to risks related to medical complications, and the effect S/A may have on nutrition, hydration and overall health and wellbeing.^2^, ^6^, ^7^ Organisational challenges include increased time for the delivery of radiation therapy and the necessity for the paediatric anaesthesia and recovery team to travel to an adult facility;^4^, ^8^, ^9^ increased costs due to the delivery of S/A; and scheduling constraints.^10^ These factors also increase health costs.^10^

As such, there is a need for interventions to reduce distress and anxiety, and support paediatric patients to remain still during radiation therapy. Our systematic review^11^ identified that very few studies have been conducted to test interventions designed to reduce psychological distress. Out of six studies, which reported significant effects, only one study reported group differences in children's reported anxiety after receiving an intervention. Our review concluded that cognitive‐behavioural approaches need to be explored further so that patients can be better supported. In our review, we also highlighted that therapeutic play may be beneficial.

An example of an intervention using such approaches was reported by McCoola et al.^12^ who evaluated the use and efficacy of behavioural therapy techniques used by the paediatric radiation therapy group in their department, 257 patient cases were included. This study identified that participants who were 3 years of age or younger continued to have the same GA requirements regardless of the behavioural interventions provided. In comparison, 78% of children aged 3–8 years‐old were provided no GA or ceased GA during treatment. Play appointments also assist children in this age group. The authors concluded that there were benefits to providing behavioural therapy resources, including play appointments, for this cohort. Another study examined the role of a certified child life specialist (CCLS) for children aged 2–12 years of age. A child life specialist is a healthcare professional who works with children, often in hospital settings, to support the child (and their family). They encourage coping skills, prepare the child for treatments and help the child express feelings. In Australia, a relevant degree is required, for example, in early childhood education. These health professionals are certified by the Child Life Certification Commission. The authors reported that adopting a CCLS model significantly reduced the frequency of daily S/A for paediatric radiation therapy patients.^10^

Looking outside of the radiation therapy setting, Gjaerde et al.^13^ conducted a scoping review on play interventions for paediatric patients in hospitals. This study highlighted that play can be used for preparation and support, developing knowledge around disease and treatment, treatment and recovery (e.g. rehabilitation and exercise) and to support adaptation to strange or new situations the children may be in.

Internationally, Holt et al.^14^ highlighted that in addition to play therapy children, may benefit from strategies such as video‐based distraction therapy and virtual reality. One example of this is described by Tennant et al.^15^ who trialled immersive virtual reality with a sample of 30 paediatric and adolescent patients receiving radiation therapy. A further study was described by Schenck et al.^16^ who developed a smartphone virtual reality game for children and adolescents receiving proton radiation therapy to reduce stress and anxiety.

However, there is a lack of health professional awareness of effective strategies to improve the experience of paediatric patients and their caregivers. It is also a challenge to know when S/A will be needed. Chiesa et al.^17^ developed a tool (The Multidimensional Assessment for Pediatric Patients in Radiotherapy (M.A.P.‐RT schedule)) to predict the need for S/A in paediatric patients and suggest that decisions around therapeutic and management procedures can be based on data after completion of this tool. This could aid planning and communication.

A systemic/organisational issue is that paediatric radiation therapy often occurs in adult settings where the environment is not developmentally adapted or considered ‘child friendly’, despite the efforts of radiation oncology staff.^5^, ^18^ This environment, including unfamiliar treatment machines and equipment, sounds and masks in medical radiation science procedures, may be anxiety provoking.^19^, ^20^, ^21^ Reducing patient anxiety around immobilisation or safety masks has been identified as an issue that also needs to be addressed in adults.^22^ Despite the attempts of individual health professionals and teams to support paediatric patients and their families, the adult medical system is not conducive to child‐centric care, and each setting needs to continually work towards ensuring paediatric patients' needs are met and support is available.

Further research is required to test interventions for paediatric radiation therapy patients. As outlined above, children may benefit from interventions such as therapeutic play, behavioural therapy and virtual reality. Research with adult cancer patients undergoing radiation therapy has also found that the provision of procedural and sensory information alongside radiation therapist communication skills training can significantly reduce adult patients' anxiety prior to treatment commencement (RT Prepare).^23^, ^24^, ^25^ However, little research has been conducted in the area of paediatric radiation therapy, where the need to communicate well with both the children and their parents is crucial as uncertainty and ambiguity can lead to increased anxiety. Children and families may benefit from a focus on procedural and sensory preparation, as well as time with radiation therapists who can recognise and respond to their emotional cues. Additionally, parents may benefit from information and support at this time, which may in turn help to reduce the children's anxiety. Families need to interpret, often complex, information and make critical decisions, and they need clear information to guide these decisions.

Importantly, there is a lack of robust research in this area, and our systematic review^11^ found that the overall quality of studies is not high. All the studies we looked at were single‐site studies, and there was little consistency in methods across studies, particularly around outcome measures. Most interventions were not theoretically driven and tended to be generic and wide‐ranging, and there is a need for longitudinal research to see if any improvements in outcome measures are sustained.

Halkett et al.'s^23^, ^24^, ^25^ RT Prepare intervention for adults started with qualitative research to explore the issues facing patients undergoing radiation therapy, and a programme of research was developed, resulting in a multi‐site, randomised controlled trial testing the intervention. The smartphone virtual reality game for children and adolescents receiving proton radiation therapy mentioned above was also developed after interviews with medical professionals and patients.^16^ Arguably, qualitative research is needed as a first step in developing theoretically and empirically driven interventions for paediatric patients undergoing radiation therapy.

## CONFLICT OF INTEREST

The authors declare no conflict of interest.
